# Effect of Celecoxib and Infliximab against Multiple Organ Damage Induced by Sepsis in Rats: A Comparative Study

**DOI:** 10.3390/biomedicines10071613

**Published:** 2022-07-06

**Authors:** Shaymaa Ramzy Senousy, Mahmoud El-Daly, Ahmed R. N. Ibrahim, Mohamed Montaser A. Khalifa, Al-Shaimaa F. Ahmed

**Affiliations:** 1 Department of Pharmacology and Toxicology, Faculty of Pharmacy, Minia University, Minia 61511, Egypt; shaymaa.ramzy@yahoo.com (S.R.S.); eldaly_m@mu.edu.eg (M.E.-D.); mmkhalifa2005@yahoo.com (M.M.A.K.); shaimaa.faissal@minia.edu.eg (A.-S.F.A.); 2 Department of Clinical Pharmacy, College of Pharmacy, King Khalid University, Abha 62529, Saudi Arabia; 3 Department of Biochemistry, Faculty of Pharmacy, Minia University, Minia 61511, Egypt

**Keywords:** celecoxib, infliximab, multiple organ damage, sepsis, antioxidant

## Abstract

In cases of sepsis, the immune system responds with an uncontrolled release of proinflammatory cytokines and reactive oxygen species. The lungs, kidneys, and liver are among the early impacted organs during sepsis and are a direct cause of mortality. The aim of this study was to compare the effects of infliximab (IFX) and celecoxib (CLX) on septic rats that went through a cecal ligation and puncture (CLP) surgery to induce sepsis. This study included four groups: sham, CLP (untreated), and CLP-treated with CLX or IFX. The administration of “low dose” CLX or IFX was performed after 2 h following the induction of sepsis. Twenty-four hours following the induction of sepsis, the rats were sacrificed and blood samples were collected to evaluate kidney, liver, and lung injuries. MDA and NOx content, in addition to SOD activity and GSH levels, were evaluated in the tissue homogenates of each group. Tissue samples were also investigated histopathologically. In a separate experiment, the same groups were employed to evaluate the survival of septic rats in a 7-day observation period. The results of this study showed that treatment with either CLX or IFX ameliorated the three organs’ damage compared to septic-untreated rats, decreased oxidative stress, enhanced the antioxidant defense, and reduced serum cytokines. As a result, a higher survival rate resulted: 62.5% and 37.5% after the administration of CLX and IFX, respectively, compared to 0% in the CLP group after 7 days. No significant differences were observed between the two agents in all measured parameters. Histopathological examination confirmed the observed results. In conclusion, CLX and IFX ameliorated lung, kidney, and liver injuries associated with sepsis through anti-inflammatory and antioxidant actions, which correlated to the increase in survival observed with both of them.

## 1. Introduction

Sepsis represents one of the most serious global clinical conditions in critical care units. A high mortality rate (between 25% and 52%) associated with sepsis correlates to multiple organ dysfunction occurring during the exaggerated immune response to infections [1]. The early affected organs during sepsis are the lungs, kidneys, and liver. Dysfunction in two or three of them is associated with the highest mortality in septic patients [2]. The exaggerated immune response to combat and contain an infection results in a “cytokine storm” [3]. Cytokines are important pleiotropic regulators of the immune response, and have a crucial role in the complicated pathophysiology of sepsis. By possessing both pro- and anti-inflammatory properties, they are capable of regulating the immune response during infection. Two phases are manifested during the course of sepsis. The first is hyperdynamic, associated with an enormous release of proinflammatory cytokines from macrophages and neutrophils, such as tumor necrosis factor (TNF)-α, interleukin (IL)-1, IL-6, and reactive oxygen species (ROS) (nitric oxide (NO), hydrogen peroxide and superoxide). The continuity of this phase leads to septic shock and multiorgan dysfunction [4,5,6]. During the second phase, a hypodynamic one, anti-inflammatory cytokines, such as IL-10, are released. Likewise, if the second phase is prolonged, a state of immunosuppression will occur, which may result in secondary infections [7] and a further worsening of the septic condition results [8]. Thus, one of the commonest strategies suggested when investigating new strategies in sepsis management is to block the activity of proinflammatory cytokines [9,10]. Besides inflammatory cytokines, cyclo-oxygenase (COX)-2 overexpression has been found to play a profound role in sepsis. It leads to the systemic dissemination of microorganisms, disturbed secretion of inflammatory cytokines and free radicals, increased vascular and intestinal permeability, development of multisystem hypoperfusion, and multiple organ dysfunction [11].

Earlier studies have investigated the effect of blockading inflammatory cytokines such as TNF-α, and others have looked at the effect of inhibiting COX-2 activity against sepsis. For example, Ozer, et al. [12] found that the survival of septic animals given infliximab (IFX), an antibody against TNF-α, as a prophylactic treatment prior to cecal ligation and puncture (CLP) surgery increased. Another study [13] showed that the administration of celecoxib (CLX), a selective COX -2 inhibitor, after CLP also resulted in a reduced mortality rate. The survival rates reported in these studies were 57% and 43%, respectively, which might be improved by changing the dose and/or timing of the treatment, factors considered to be critical in the management of sepsis [14,15,16].

Thus, this study was conducted with two aims: 1—To investigate the effects of post-treatments with different doses of these agents against sepsis. 2—To compare the efficacy of each agent in a single study, focusing on their role as early interventions rather than protective agents. The survival rate, as well as the extent of multiple organ damage in septic rats, was used to perform this comparison. The model used in the present study for the induction of sepsis is CLP. The CLP model is the most widely applied model for the experimental induction of sepsis [17]. Many requirements can be met through performing CLP: induction of polymicrobial sepsis, subsequent release of endotoxins, and simplicity. All of these criteria make CLP one of the most clinically relevant models for the induction of sepsis [18].

## 2. Materials and Methods

### 2.1. Experimental Animals and Design

Female albino Wistar rats (200 ± 20 g) were obtained from Nahda University at BeniSuef (NUB) Animal House (Beni-Suef, Egypt). Before performing the experiment, for one week and throughout the whole experiment, all rats were accommodated in plastic cages under a 12 h dark–light cycle at room temperature (25 ± 2 °C) with a standard diet and water supplied ad libitum. All procedures reported in this experiment were according to the international ethical guidelines and the National Institutes of Health Guide concerning the Care and Use of Laboratory Animals, and were approved by the Commission on Ethics of Scientific Research, Faculty of Pharmacy, Minia University (code number of the project: ES12/2020).

Celecoxib was obtained from Pfizer, USA. IFX was obtained from Janssen Pharmaceuticals, USA. Thirty rats were randomly allocated into 4 groups: sham (*n* = 6), CLP (*n* = 12), CLP/CLX (0.1 mg/kg, p.o) [19], 2 h following the CLP surgery (*n* = 6), and CLP/IFX (5 mg/kg, s.c.) [20], 2 h following CLP induction (*n* = 6).

### 2.2. Induction of Sepsis by CLP

Polymicrobial sepsis was induced by performing CLP, the gold-standard model of sepsis. To keep the severity of sepsis consistent, 75% of the cecum was ligated. Then, the ligated part of the cecum was thoroughly punctured twice with an 18-gauge needle. All animals underwent the same procedure, while the ligation and puncture steps were not performed in the sham group. [21]. The animals did not receive any analgesics, as they interfere with measured parameters. Efforts were made to reduce the suffering of the animals, and all rats were checked for signs of severe illness twice daily and euthanized if their health scores were above a certain value according to Morton and Griffiths [22].

### 2.3. Survival Experiment

The same groups reported previously were allocated for the survival study. Thirty-two rats were randomly divided into four groups (*n* = 8 per each) as follows: sham group, CLP group, CLX-treated septic group, and IFX- treated septic group. All rats were observed for seven days to record mortality [23].

### 2.4. Blood Collection, Tissue Isolation, and Preparation

Sodium thiopental (50 mg/kg) was used to anesthetize the rats 24 h following the induction of sepsis. A cardiac puncture was performed to collect blood samples and then left for 10 min before centrifugation at 2500 rpm for 10 min. Portions of the liver medial lobe, the left kidney, and the right lower lobe of the lung were rapidly dissected and dried using filter paper. The other kidney, liver portions, and left lung were flash-frozen in liquid nitrogen and stored at −20 °C for other measurements [24]. Prior to those measurements, all tissues were homogenized using a motor-driven homogenizer (Tri-R Stir-R homogenizer, Tri-R Instruments, Inc., Rockville Centre, NY, USA) in an ice bath for 10 min (in phosphate-buffered saline (PBS), PH 7.4 in 5% W/V). Analysis of oxidative stress markers and antioxidant defense activity was performed in the supernatant separated from the homogenates after centrifugation for 15 min at 4000 rpm.

### 2.5. Histopathological Examination

Lung, kidney, and liver tissues were processed for standard hematoxylin and eosin (H&E) staining, then fixed in neutral buffered formalin solution (10%) to be examined with electric light microscope [25].

Lung, liver, and kidney tissues from each group were pathologically assessed blindly. Histopathological changes in lung tissues were determined according to infiltration of inflammatory cells, hemorrhage, congestion, and edema on a scale ranging from 1 to 4 as follows: 0, absent; 1, light; 2, moderate; 3, strong; 4, intense. The lung injury score was recorded as the mean of the scores for each individual parameter. The percentages of cellular necrosis in renal tubules were assessed for the evaluation of the kidney injury score as follows: 0 = none, 1 = 0–20%, 2 = 20–50%, 3 = 50–70%, 4 = more than 70% of renal tubules were necrotic. Ten fields at least were examined for each animal [26].

Congestion, edema, infiltration of polymorphonuclear leukocytes and monocytes, and necrosis were the four criteria determined for the assessment of the level of liver injury on a scale from 0 to 4. Congestion scored 1; edema scored 2. Infiltrations of polymorphonuclear leukocytes and monocytes scored 3. Necrosis scored 4. The sum of the previously mentioned scores resulted in a total score from 0 to 10 [27].

### 2.6. Assessment of Microvascular Permeability and Pulmonary Edema

In order to assess microvascular permeability of the lung and pulmonary edema, the lung was intubated and lavaged with cold PBS (0.5 mL four times) immediately after the collection of blood via cardiac puncture so the total leukocytic cells in the bronchoalveolar lavage fluid (BALF) could be counted [28]. The cell pellet obtained after centrifugation of BALF (at 1000 rpm for 10 min at 4 °C) was resuspended (in 0.5 mL PBS) to count the total number of the cells using a hematology analyzer (Mindray Bc-20 s Auto Hematology Analyzer) [29].

Following that step, the lungs were collected, washed, and the right upper lobe was dissected and weighed using a sensitive electric scale (analytical balance 220/C/2, RADWAG, Radom, Poland). The right upper lobe was then dried in an oven at 80 °C for 24 h. The dried lobe was weighed using the same balance. The lung wet/dry (W/D) weight ratio gave an indication of pulmonary edema [30].

### 2.7. Assessment of Total Protein in BALF

The supernatant of the BALF was used to determine total protein concentration through the colorimetric method (endpoint) according to George and KINGSLEY [31], which was recommended by the manufacturer (BioMed kit, Cairo, Egypt).

### 2.8. Assessment of Kidney Function

Commercial kits were purchased from (Biodiagnostic, Egypt) to measure serum levels of creatinine (CR) and blood urea nitrogen (BUN) according to the methods that were originally described by [32,33], respectively. Cystatin C was also measured as a reliable marker for kidney injury, which is not affected by gender, age, or muscle mass [34]. According to the manufacturer’s instructions, a commercial ELISA kit was purchased from Elabscience Biotechnology (Houston, TX, USA) to assess serum cystatin C.

### 2.9. Assessment of Liver Function

A previously described methodology [35] was used to assess serum alanine aminotransferase (ALT) and aspartate aminotransferase (AST) levels using commercial kits obtained from Biodiagnostic (Cairo, Egypt).

### 2.10. Assessment of Oxidative Stress Markers and Antioxidant Defense Activity in Lung, Liver, and Kidney Homogenates

Lipid peroxidation was determined according to the tissue levels of malondialdehyde (MDA), which were assessed colorimetrically following the previously described methodology in [36] for all tissue homogenates. Total nitrate content was measured colorimetrically using the Griess reaction [37], in which nitrates are reduced to nitrites by cadmium.

For assessment of the antioxidant defense activity, superoxide dismutase (SOD) activity was measured spectrophotometrically according to the method described in [38] with a slight modification based on the fact that the autoxidation of pyrogallol is inhibited by SOD, and is generally defined as the amount of SOD enzyme that inhibits the autoxidation of pyrogallol by 50%. Additionally, the tissue levels of reduced glutathione (GSH) were colorimetrically determined following the method described previously in [39] at 412 nm.

### 2.11. Determination of Serum Inflammatory Markers

Available commercial ELISA kits were purchased to assess serum inflammatory cytokines (TNF-α, IL-6, IL-1β) levels after constituting a standard curve to measure serum concentration in different serum samples, according to the manufacturer’s instructions (Elabscience Biotechnology, Houston, TX, USA) at 450 nm, spectrophotometrically.

### 2.12. Statistical Analysis

GraphPad Prism (version 7.0; San Diego, CA, USA) was used for statistical analysis of the data. Multiple comparisons were performed using a one-way ANOVA test followed by a Tukey–Kramer post hoc test. All reported results were expressed as mean ± S.E.M. Survival analysis was performed through the logrank (Mantel–Cox) test. Results were considered statistically significant at *p*-values less than 0.05. If the values of the Pearson correlation coefficient (r) that was used to calculate the correlation between the parameters of the study were less than |0.19|, correlation was considered very weak. If r was between |0.2| and |0.39|, the correlation was considered weak. Values of (r) between |0.4| and |0.59| referred to moderate correlation. Correlation was considered strong if the value of r was between |0.6| to |0.79|, while the correlation was considered very strong if r > |0.79| [40].

## 3. Results

### 3.1. Celecoxib and Infliximab Improve Survival in Septic Rats

**Figure 1:**
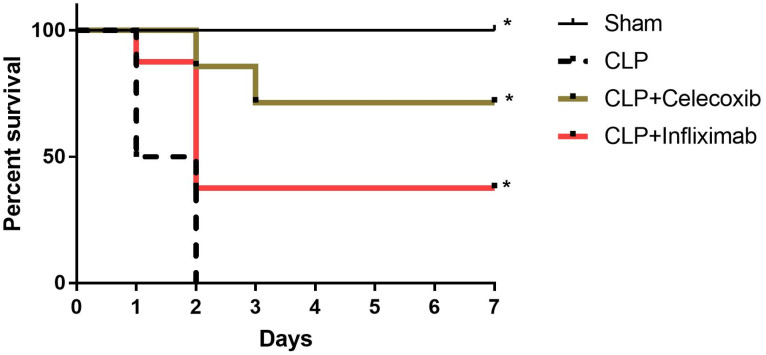
The effects of the induction of sepsis and treatment with CLX and IFX on survival. Cecal ligation and puncture (CLP) surgery resulted in a decrease in the survival rate, reaching 0 % survival at the end of day 2, while celecoxib (CLX) (0.1 mg/kg, p.o), which was administered to the rats 2 h after CLP, improved survival at the end of the study by 50%. Infliximab (IFX) (5 mg/kg, s.c), which was injected into the rats 2 h following induction of sepsis, increased survival by the end of the seventh day, reaching 37.5% compared to the septic untreated group. The survival rate of rats in the sham group of the study was 100%. Rats were monitored for 7 days. Data are described as a percentage of the survival of the rats (*n* = 8 rats per group). * refers to a significant difference at *p* < 0.05 compared to the CLP group.

The rats were monitored for seven days after the induction of sepsis every 24 h. After the first day, the induction of sepsis resulted in 50% mortality, while treatment with CLX (0.1 mg/kg, p.o) showed significant protection against sepsis, which was 0% mortality within the first day. In addition, IFX (5 mg/kg, s.c) resulted in an 87.5% increase in survival at the end of the first day. By the end of the second day, the CLP group showed 0% survival, while at the end of the study (the end of the seventh day), treatment with either CLX or IFX significantly (*p* < 0.05) enhanced the survival of the septic rats (62.5% and 37.5%, respectively), with no significant difference between them. No death events were observed in the sham group. (Figure 1).

### 3.2. Celecoxib and Infliximab Ameliorate CLP-Induced Oxidative Stress

The results, presented in Table 1, Table 2 and Table 3, show that the induction of sepsis significantly (*p* < 0.05) reduced the tissue levels of GSH compared to the non-septic rats. On the other hand, treatment with CLX significantly (*p* < 0.05) elevated its level compared to the CLP group. Similarly, treatment with IFX significantly (*p* < 0.05) prevented CLP-mediated GSH depletion in these tissues.

Renal, hepatic, and pulmonary SOD activity were severely affected due to CLP induction in comparison to the sham group. However, the administration of either CLX or IFX significantly (*p* < 0.05) enhanced SOD activity compared to the septic, non-treated rats.

Kidney, liver, and lung MDA levels significantly (*p* < 0.05) increased after the induction of sepsis compared to the sham group. Moreover, CLX administration significantly (*p* < 0.05) reduced the raised levels of MDA. Similarly, IFX injection significantly (*p* < 0.05) decreased the tissue levels of MDA compared to the CLP group. Renal, hepatic, and pulmonary NOx levels were significantly (*p* < 0.05) boosted after the induction of sepsis when compared to the sham group. The administration of either CLX or IFX significantly (*p* < 0.05) reduced the evident rise seen in the CLP group. No significant difference was observed in the effect of both drugs on the tissue levels of GSH, MDA, or the NOx and SOD activity in all examined tissues. (Table 1, Table 2 and Table 3).

### 3.3. Celecoxib and Infliximab Attenuate CLP-Induced Inflammatory Signals

**Figure 2:**
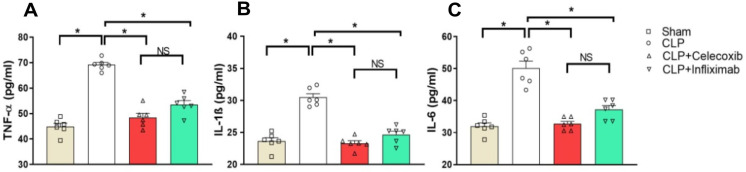
CLX and IFX ameliorate serum inflammatory profiles in septic rats. The bar charts show the effects of CLX and IFX on serum cytokines. (**A**) TNF-α, (**B**) IL-1β, (**C**) IL-6. ANOVA was used to analyze the results, followed by the Tukey–Kramer test for multiple comparisons. For each group, *n* = 6. CLP: cecal ligation and puncture, CLX: celecoxib, IFX: infliximab. * denotes significant at *p* < 0.05.

As shown in Figure 2, the serum levels of TNF-α increased significantly (*p* < 0.05) following the induction of sepsis. On the other hand, treatment with either CLX or IFX showed a significant (*p* < 0.05) reduction in the raised levels of TNF-α compared to the septic untreated rats. Additionally, the serum levels of IL-1β significantly (*p* < 0.05) increased after performing the CLP procedure. Such high levels were significantly (*p* < 0.05) reduced following the administration of either CLX or IFX 2 h after the induction of sepsis. Similarly, following the CLP technique, a significant (*p* < 0.05) rise was observed in the serum levels of IL-6 in the septic untreated group compared to sham-operated rats. However, rats treated with either CLX or IFX showed a significant reduction (*p* < 0.05) in the serum levels of IL-6 compared to the septic untreated rats. No significant difference was observed in the effect of both drugs on the serum levels of TNF-α, IL-1β, and IL-6.

### 3.4. Protective Effect of Celecoxib and Infliximab against Sepsis-Induced Acute Lung Injury (ALI)

**Figure 3:**
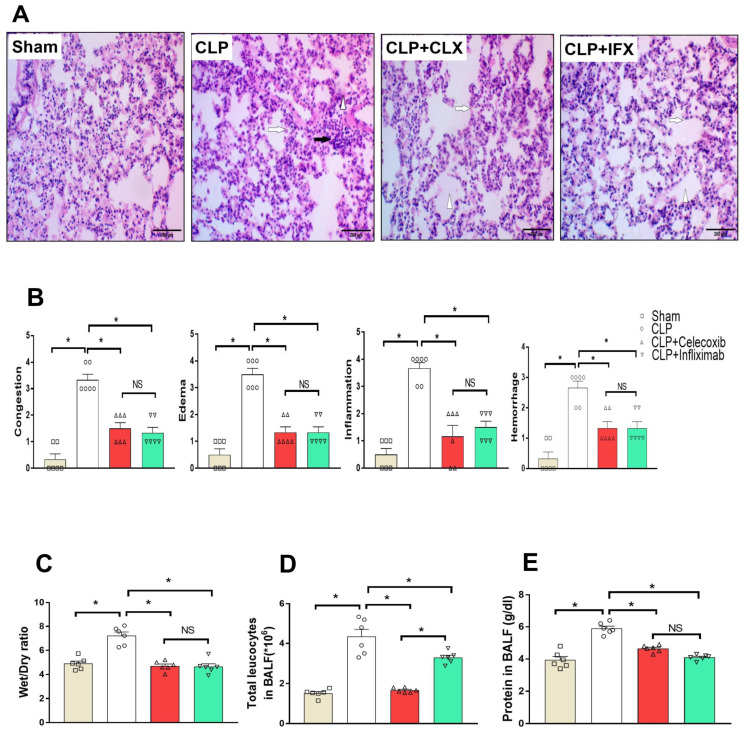
Effect of CLP and treatment with CLX and IFX against sepsis-induced ALI. (**A**) Photomicrographs showing the effect of CLX and IFX administration on sepsis-induced histopathological lesions in rat lungs (H&E stain, 200×). The sham group revealed normal alveoli with intact alveolar membranes and alveolar spaces with intervening bronchioles. A thickened alveolar membrane in the CLP group was evident due to edema and inflammatory infiltrate (white arrow). Dilated and congested alveolar spaces with focal areas of alveolar membrane damage (arrowhead), interstitial tissue congestion, and areas of inflammatory infiltrates were formed mainly from alveolar macrophages and lymphocytes (black arrow). The CLP/CLX and CLP/IFX groups decreased such injuries following the induction of sepsis, which was apparent as multiple alveoli with intact alveolar membranes without rupture, inflammatory infiltrates (arrow), non-congested interstitial and empty alveolar spaces without hemorrhage or edema (arrowhead). (**B**) Analysis of lung injury score. (**C**) W/D weight ratio of lung tissue from different groups. (**D**) The total number of leukocytes in BALF from different groups. (**E**). Total protein content in BALF from different groups. Each bar represents the mean ± SEM; *n* = 6 for all groups. * denotes significant difference at *p* < 0.05.

Figure 3 shows H&E-stained lung sections and the histopathological scores of lung tissues. The difference between the sham group and the CLP group was apparent in the form of the normal alveoli with intact alveolar membranes and alveolar spaces with intervening bronchioles, which was evident upon the examination of lung tissues from the sham group. On the other hand, alveolar membranes thickened because of the edema, and inflammatory infiltrates were observed in lung tissues from rats subjected to CLP surgery. Dilated and congested alveolar spaces with focal areas of alveolar membrane damage, interstitial tissue congestion, and areas of inflammatory infiltrates were also observed in the CLP group, and these were composed mainly of alveolar macrophages and lymphocytes. In the CLP/CLX and CLP/IFX groups, multiple alveoli with intact alveolar membranes, without any ruptures or inflammatory infiltrates, were apparent, which reflects their protective effects. In addition, their ameliorative effects were confirmed in the form of non-congested, interstitial, and empty alveolar spaces, without hemorrhage or edema (Figure 3A).

Histological change scores in lung tissues were significantly (*p* < 0.05) high in the CLP group compared to the sham group. Lung tissue scores in the CLP/CLX and CLP/IFX groups significantly (*p* < 0.05) decreased compared to the CLP group (Figure 3B). There was no significant (*p* < 0.05) difference between the effects of both drugs on lung injury scores. The wet/dry weight ratio, as a detector of lung tissue inflammation, increased significantly (*p* < 0.05) in the CLP group. Treatment with CLX and IFX significantly (*p* < 0.05) reduced lung the wet/dry weight ratio compared to the CLP group (Figure 3C). In addition, we investigated the total number of leukocytes in BALF, which increased significantly (*p* < 0.05) in the CLP group compared to the sham group (4.31 ± 0.31 vs. 1.52 ± 0.13 for CLP and sham, respectively). As shown in Figure 3D, the amount of these cells decreased significantly (*p* < 0.05) in CLX- and IFX- treated septic rats. Moreover, we detected the total protein content in BALF, as shown in Figure 3E. The total protein content significantly (*p* < 0.05) increased in septic untreated rats compared to the sham-operated rats. Treatment of the CLP group with CLX and IFX resulted in a marked (*p* < 0.05) reduction in the total protein content. There was no significant difference between the CLX and IFX effects on the wet/dry weight and total protein content, but a significant difference among them was observed in the total number of leukocytic cells in BALF, as shown in Figure 3D.

### 3.5. Protective Effect of Celecoxib and Infliximab against Sepsis-Induced Acute Hepatic Injury (AHI)

**Figure 4:**
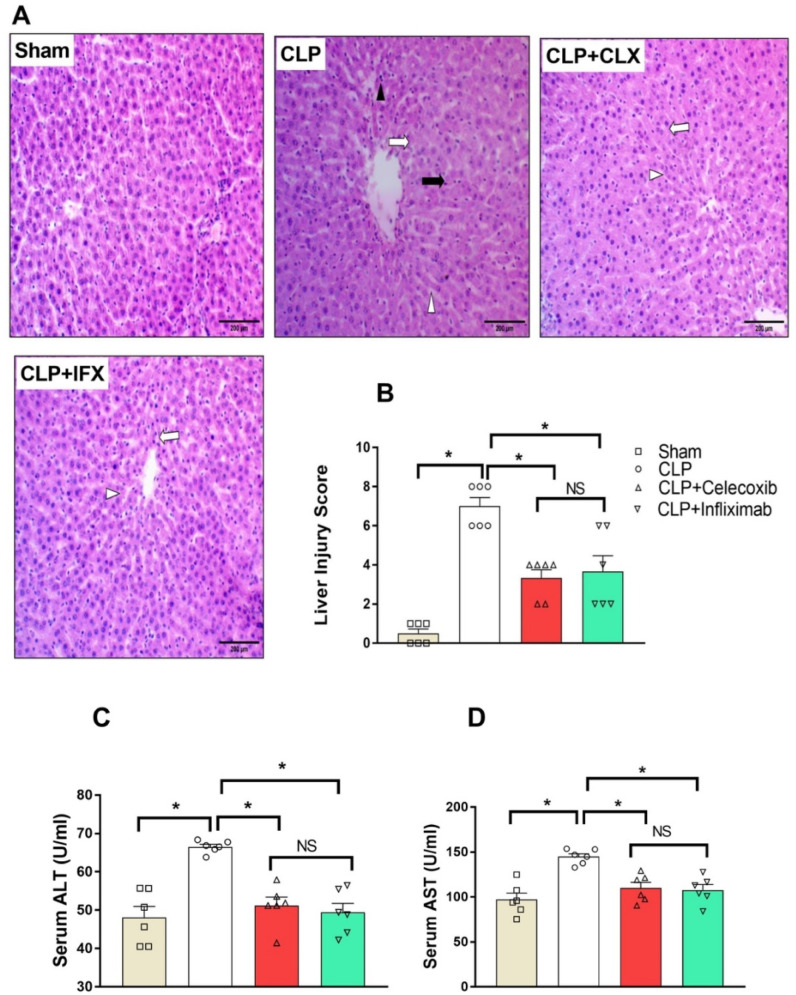
Effect of CLP and treatment with CLX and IFX against sepsis-induced AHI. (**A**) Photomicrographs showing the effect of CLX and IFX administration on sepsis-induced liver injury (H&E stain, 200×). The sham group showed non-congested central veins and normally arranged hepatocyte cords with normal intervening non-congested sinusoids. The CLP group showed dilated congested central veins surrounded by peripherally arranged hepatocyte cords showing mild to moderated vacuolar degeneration in the form of faint eosinophilic cytoplasm with a central basophilic nucleus (white arrow); hepatocytes are separated by dilated and congested hepatic sinusoids (white arrowhead). Kupffer cell prominence with oval to triangular-shaped nuclei (black arrow) and chronic inflammatory cellular infiltrates in the form of clusters of lymphocytes and macrophages (black arrowhead) was observed. The CLP/CLX and CLP/IFX groups showed marked protection against the effect of sepsis, which was apparent in the form of a non-congested central vein with peripherally arranged hepatocytes with eosinophilic cytoplasm and a central nucleus without fatty vacuoles or swelling (arrow). Hepatocyte cords were separated by non-congested sinusoidal spaces (arrowhead), with no inflammatory cellular infiltrates and no necrotic focal regions. (**B**) Analysis of liver injury score. (**C**) Effect of different groups on serum ALT. (**D**). Effect of different groups on serum AST. Each value represents the mean ± SEM; *n* = 6 for all groups. * denotes significant at *p* < 0.05.

H&E-stained liver sections are shown in Figure 4. The CLP group showed a dilated and congested central vein surrounded by peripherally arranged hepatocyte cords showing mild to moderate vacuolar degeneration in the form of faint eosinophilic cytoplasm and a central basophilic nucleus. Hepatocytes were separated by dilated and congested hepatic sinusoids. Such manifestations were completely different from the non-congested central vein and the normally arranged hepatocyte cords, with normal intervening non-congested sinusoids, observed in the sham group. Additionally, in the CLP group, Kupffer cell prominence, with oval to triangular-shaped nuclei and chronic inflammatory cellular infiltrates in the form of clusters of lymphocytes and macrophages, were also observed. The CLP/CLX and CLP/IFX groups revealed marked amelioration in the effect induced following CLP surgery, which was observed in the form of a non-congested central vein with peripherally arranged hepatocytes with eosinophilic cytoplasm and a central nucleus without fatty vacuoles or swelling. Additionally, the hepatocyte cords were separated by non-congested sinusoidal spaces. Finally, neither inflammatory cellular infiltrates nor necrotic focal regions were observed in either group (Figure 4A).

Histological change scores in liver tissues were significantly (*p* < 0.05) high in the CLP group compared to the sham group. Liver tissue scores in the CLP/CLX and CLP/IFX groups were significantly reduced compared to the CLP group (Figure 4B). There was no significant (*p* < 0.05) difference between the effects of both drugs on liver injury scores. The liver function parameters ALT and AST were markedly elevated in septic rats compared to sham animals (66.53 ± 0.80 and 144.9 ± 4.19 in the CLP group vs. 48.15 ± 3.26 and 97.33 ± 7.04 in the sham group for ALT and AST, respectively). On the other hand, the administration of CLX and IFX significantly (*p* < 0.05) inhibited such elevation (51.23 ± 3.49 and 49.43 ± 2.88 for ALT, respectively). Similarly, the administration of CLX and IFX significantly (*p* < 0.05) inhibited that rise (110.80 ± 5.32 and 107.80 ± 5.19 for AST, respectively) (Figure 4C,D). There was no significant difference between the effects of CLX and IFX on serum ALT or serum AST levels.

### 3.6. Protective Effect of Celecoxib and Infliximab against Sepsis-Induced Acute Kidney Injury (AKI)

**Figure 5:**
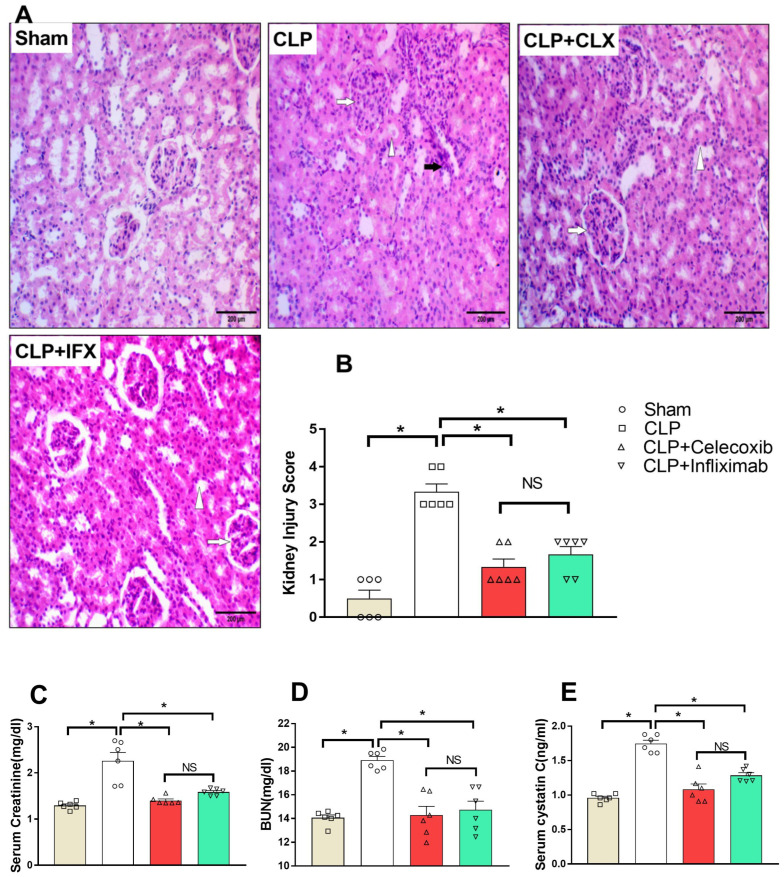
Effect of CLP induction and treatment with CLX and IFX against sepsis-induced AKI. (**A**). Photomicrographs showing the effect of CLX and IFX administration on sepsis-induced kidney injury (H&E stain, 200×). The sham group showed normal renal glomeruli and tubules. The CLP group showed injuries of the glomerulus (white arrow) in the form of congested and fibrosed glomerulus capillaries, mesangial cell proliferation, and narrow to obliterated bowman’s spaces. Tubular injury (arrowhead) came in the form of tubular edema, intratubular casts, and focal areas of tubular damage. In the interstitial tissue (black arrow), edema and chronic inflammatory cellular infiltrates were in the form of lymphocytic aggregates. The CLP/CLX and CLP/IFX groups showed marked protection against the effect of sepsis, which was apparent in the form of the normal structure of the glomerulus, without congestion or mesangial cell proliferation (arrow). Normal tubular structures (arrowhead) were without edema, tubular casts, or destruction. Interstitial tissue was thin and free of congestion or inflammation. (**B**). Analysis of kidney injury score. (**C**). Effect of different groups on serum CR. (**D**). Effect of different groups on serum BUN. (**E**). Effect of different groups on serum cystatin (**C**). Each value represents the mean ± SEM; *n* = 6 for all groups. * denotes significant difference at *p* < 0.05.

As shown in the H&E-stained kidney sections (Figure 5), normal renal glomeruli and tubules in the sham group were evident. On the other hand, glomerulus injury was apparent in the CLP group in the form of congested and fibrosed glomerulus capillaries, mesangial cell proliferation, and narrow to obliterated bowman’s spaces. A tubular injury was also observed in the CLP group as tubular edema, intratubular casts, focal areas of tubular damage, and interstitial tissue. Tissue edema and chronic inflammatory cellular infiltrate were in the form of aggregates of lymphocytes. Interestingly, the administration of either CLX or IFX resulted in marked protection against the effects that were evoked after the CLP surgery. Such protection was apparent in the form of the normal structure of the glomerulus, which had no congestion or mesangial cell proliferation. In addition, normal tubular structures, with no edema, tubular casts, or destruction, and interstitial tissue, thin and free from congestion and inflammation, confirm the ameliorative effect of either CLX or IFX (Figure 5A). Histological change scores in kidney tissues were significantly (*p* < 0.05) high in the CLP group compared to the sham group. Kidney tissue scores in the CLP/CLX and CLP/IFX groups were markedly reduced compared to CLP group (Figure 5B). There was no significant (*p* < 0.05) difference between the effects of both drugs on renal injury scores.

Serum creatinine, BUN, and cystatin C were measured to reflect the kidney function following CLP induction and the administration of CLX and IFX. CLP induction resulted in a marked (*p* < 0.05) elevation in serum kidney function parameters in septic rats compared to sham animals. On the other hand, the administration of CLX significantly inhibited that elevation. Similarly, the administration of IFX significantly inhibited that elevation (Figure 5C–E). There was no significant difference between the effects of both drugs on kidney injury score or any of the measured kidney function parameters.

### 3.7. Analysis of Correlation between Different Study Parameters and Lung Injury Score

**Figure 6:**
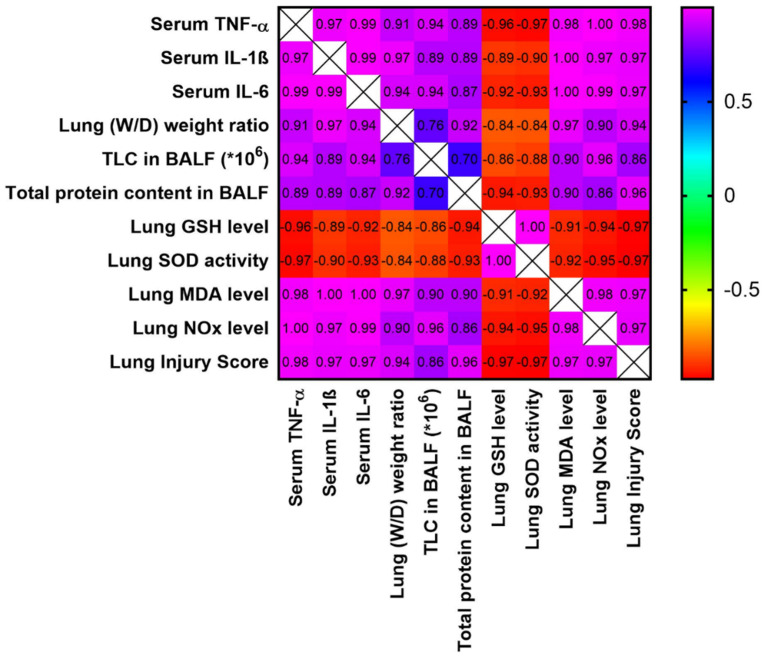
Correlation matrix for different parameters included in the study and the lung injury scores. The correlation coefficient used for analysis is the Pearson correlation coefficient (r). Positive values of r indicate positive correlation, while negative values indicate negative correlation. The correlation was considered strong if the value of r was between |0.6| to |0.79|, while the correlation was considered very strong if r > |0.79|. The range of the colors of the scale on the right, from violet to red, refers to r values from +1 to −1. IL-1β: interleukin-1b; IL-6: interleukin-6; TNF-α: tumor necrosis factor-α; BALF: bronchoalveolar lavage; TLC: total leukocytic count; (W/D): wet/dry.

The data shown in Figure 6 demonstrate the correlation between different study parameters and the lung injury scores. As shown, the lung injury scores have a very strong positive correlation with wet/dry weight, total protein content, and the total leukocytic count in BALF. Additionally, it shows a very strong positive correlation with serum inflammatory markers (TNF-α, IL-1β, and IL-6) and the oxidative stress markers in the examined pulmonary tissues (MDA and NOx). Additionally, it shows a very strong negative correlation with the pulmonary GSH levels and SOD activity. A strong positive correlation was observed between the total leukocytic counts in BALF and total protein content. A strong positive correlation was also observed between the total leukocytic counts in BALF and the W/D weight ratio.

### 3.8. Analysis of Correlation between Different Study Parameters and Liver Injury Score

**Figure 7:**
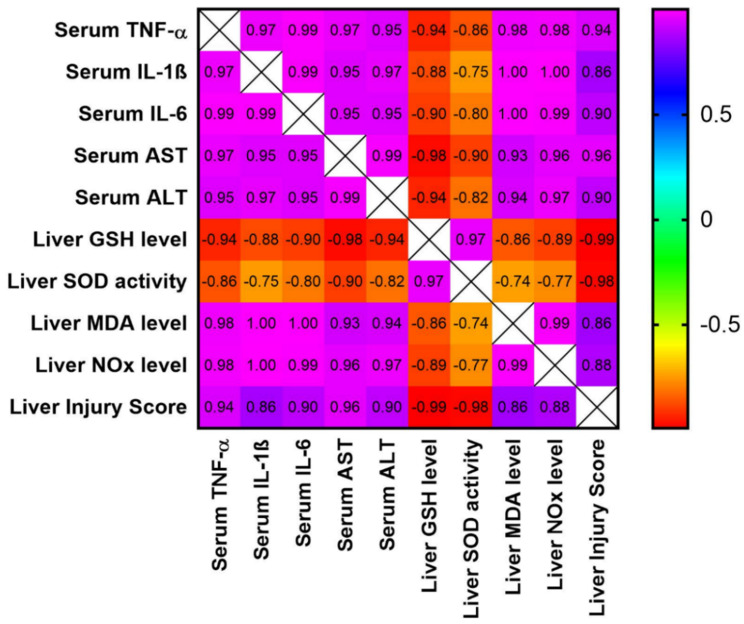
Correlation matrix for different parameters included in the study and the liver injury scores. The correlation coefficient used for analysis is the Pearson correlation coefficient (r). Positive values of r indicate positive correlation, while negative values indicate negative correlation. The correlation was considered strong if the value of r was between |0.6| to |0.79|, while the correlation was considered very strong if r > |0.79|. The range of the colors of the scale on the right, from violet to red, refers to r values from +1 to −1. IL-1β: interleukin-1b; IL-6: interleukin-6; TNF-α: tumor necrosis factor-α; ALT: alanine aminotransferase; AST: aspartate aminotransferase.

The data shown in (Figure 7) demonstrate the correlation between different study parameters and the liver injury scores. As shown, the liver injury scores have a very strong positive correlation with liver function parameters (serum ALT and AST), serum inflammatory markers (TNF-α, IL-1β and IL-6), and oxidative stress markers in the examined hepatic tissues (MDA and NOx). Additionally, it shows a very strong negative correlation with the hepatic GSH levels and SOD activity.

### 3.9. Analysis of Correlation between Different Study Parameters and Kidney Injury Score

**Figure 8:**
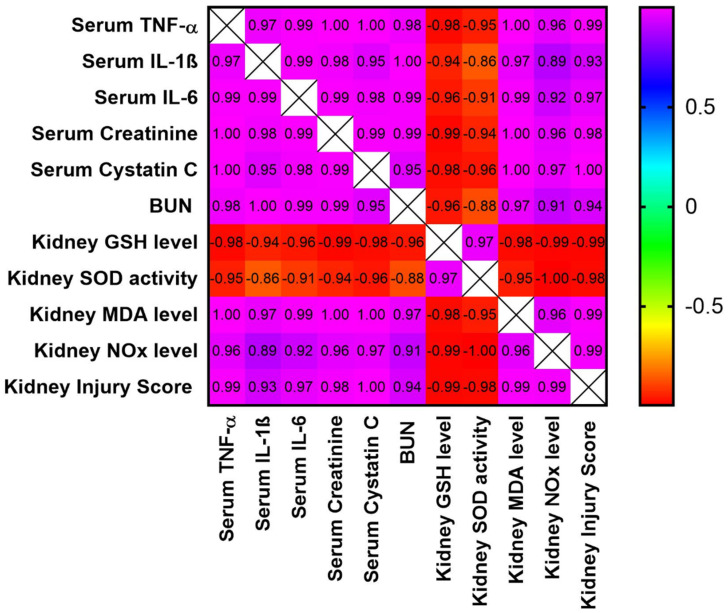
Correlation matrix for different parameters included in the study and kidney injury scores. The correlation coefficient used for analysis is the Pearson correlation coefficient (r). Positive values of r indicate positive correlation, while negative values indicate negative correlation. The correlation was considered strong if the value of r was between |0.6| to |0.79|, while the correlation was considered very strong if r > |0.79|. The range of the colors of the scale on the right, from violet to red, refers to r values from +1 to −1. IL-1β: interleukin-1b; IL-6: interleukin-6; TNF-α: tumor necrosis factor-α; BUN: blood urea nitrogen.

The data shown in Figure 8 demonstrate the correlation between the different study parameters and the kidney injury scores. As shown, the kidney injury scores have a very strong positive correlation with kidney function parameters (serum creatinine, BUN, and serum cystatin C), serum inflammatory markers (TNF-α, IL-1β, and IL-6), and oxidative stress markers in the examined renal tissues (MDA and NOx). Additionally, it shows very strong negative correlation with the renal GSH levels and SOD activity.

## 4. Discussion

Despite therapeutic advances over the last two decades, the high mortality rate associated with sepsis confirms why sepsis is considered a challenging clinical obstacle in intensive care medicine [41,42]. Such a situation confirms the existence of a knowledge gap in sepsis research, which requires ongoing research. This study was established to investigate the protective effect following the administration of CLX (0.1 mg/kg, p.o) and IFX (5 mg/kg, s.c.) to septic rats, and to compare the effects of both drugs. The original hypothesis was that CLX and IFX would protect against sepsis through their anti-inflammatory and antioxidant effects, which were speculated to involve the modulation of two pathways that are known to be upregulated during sepsis; the COX-2 and TNF-α pathways, respectively. Previous studies have investigated the effect of CLX or IFX alone, but we aimed to compare the efficacy of both drugs in a single study. We used doses smaller than the previous studies and administered our drugs as treatments after the induction of sepsis. Because previous reports showed a beneficial effect in anti-cytokine combination [43], we attempted to test the effect of a combination of CLX and INF in rats subjected to CLP, but, unfortunately, a high mortality rate was observed in this group, indicating adverse outcomes in their combination (Supplementary Materials).

Thus, the first aim of this study was to investigate the protective effect of CLX and IFX on sepsis-associated mortality rates. The results showed that no animals survived by the end of the second day following CLP surgery, which agrees with previous studies [44,45]. The cytokine storm associated with sepsis and septic shock is one of the direct causes of the observed high mortality [46]. In our study, septic untreated rats showed high serum levels of TNF-α, IL-1β, and IL-6. In addition, strong evidence confirms the role of oxidative stress in the pathogenesis of sepsis-induced acute lung, liver, and kidney injuries, which also contributes to the high mortality rates observed in sepsis [47,48,49,50,51]. As a result of the inflammatory response and oxidative stress, multiple organ dysfunction occurs, resulting in death. Nearly 40% of septic patients develop ALI. The lung is considered one of the earliest affected organs during sepsis [52]. In addition, the kidney is one of the commonest targets of the sequelae of sepsis; more than half of patients with sepsis or septic shock suffer from AKI [53,54]. Another target of the host response is the liver; about 46 percent of patients with sepsis have concomitant hepatic dysfunction, which has been associated with higher 28-day mortality [2].

In the present study, elevated serum levels of creatinine, cystatin C, and BUN emphasized the renal injury associated with sepsis. In addition, high serum levels of ALT and AST confirmed the acute hepatic injury associated with sepsis. Such results are in accordance with previous studies [55,56]. ALI was also observed as elevated lung wet/dry weight ratio, increased BALF protein output, and total leukocyte count. Our results agree with the findings of previous studies. The induction of sepsis by Ibrahim, Moussa, Bayoumi and Ahmed [26] via the CLP model showed the same signs of acute lung injury in addition to the elevated serum levels of creatinine and BUN, which indicated acute kidney injury. Furthermore, such injuries were confirmed by histopathological examinations of the organs affected.

In the study performed by Ozer et al. [12], the administration of IFX (7 mg/kg, i.p.) 24 h prior to CLP surgery increased the survival rate to 57% in comparison to our results, which showed that the administration of IFX (5 mg/kg, s.c.) 2 h following the induction of sepsis improved survival to 37.5%. This diminished survival can be explained by the lower dose that we used. Furthermore, our study was performed as a treatment rather than a prophylaxis.

Similar results have been observed in a rat model of experimental colitis in which the lower doses of IFX (5 mg/kg, s.c) showed more favorable histological results than the higher doses [20]. Our study provides evidence that posttreatment with IFX seems to improve survival in CLP-induced septic rats as a result of preventing multiple organ failure.

To gain an insight into the reason for such marked increases in the survival rate observed after the administration of IFX, we investigated the effect of IFX administration on ALI, AHI, and AKI, which are associated with sepsis. Our study showed that IFX reduced lung edema, protein content, and the number of leucocytes in BALF, which are characteristics of acute lung injury [57]. Such effects were confirmed in the histological analysis of lung tissue. Similarly, a histopathological examination of the kidney confirmed that the injury associated with CLP induction in the renal tissues markedly decreased in the IFX-treated group. These findings were supported by an ameliorative effect of IFX on the CLP-induced elevation of cystatin C, BUN, and CR levels, which are very characteristic during the development of sepsis [58,59]. The administration of IFX also antagonized acute hepatic injuries, manifested as reductions in AST and ALT serum levels, confirmed histopathologically in the examined hepatic tissues.

The excessive release of TNF-α, IL-1β, and IL-6 leads to a massive release of other proinflammatory cytokines and ROS production, which ends with cellular injury and apoptosis [60,61]. As evident from the results section, the protective effects of IFX against ALI, AHI, and AKI were correlated with the evident anti-inflammatory and antioxidant properties of that agent and, subsequently, a higher survival rate was observed. In a previous study performed by Aydin, et al. [62], IFX reduced serum levels of TNF-α and MDA and increased SOD activity, thus decreasing small intestinal injury. Another study reported that the reduction of NO in renal tissues contributed to the protective effect of IFX against methotrexate-induced nephrotoxicity [61]. The suppression of the genetic expression of NF-kB, TNF-α, IL-1β, and IL-6 in vas deferens tissues was suggested as the protective mechanism of IFX against ejaculatory dysfunction [63]. Additionally, in a previous study performed by Altintas, et al. [64], IFX reduced lipid peroxidation in pulmonary tissues and increased the SOD activity and tissue levels of GSH, thus protecting against bleomycin-induced lung fibrosis. Such antioxidant effects have also been reported previously in a rat model of intestinal ischemia/reperfusion (I/R) [65]. Moreover, Guzel, et al. [66] reported the ability of IFX to reduce ALI in an experimental model of intestinal I/R and suggested that such an effect was due to its ability to reduce the tissue levels of MDA and increase SOD activity in lung tissues.

In a study performed by Ozer, Goktas, Kilinc, Bariskaner, Ugurluoglu and Iskit [13], the administration of CLX (0.5 mg/kg, p.o) 2 h post-CLP surgery resulted in a 43% survival rate in septic rats at the fourth day. In the present study, CLX was administered after the induction of sepsis in a dose of 0.1 mg/kg, p.o, leading to a 62.5 % increase in survival within 7 days, which is higher than that achieved by the former study despite the smaller dose that was used in the current study. Such a difference in survival rates between the two studies could be explained by the higher dose used by Ozer, Goktas, Kilinc, Bariskaner, Ugurluoglu and Iskit [13], which could contribute to the excessive inhibition of COX-2, affecting the necessary level of COX-2 activity. As a consequence of the overexpression of COX-2 during inflammation and organ damage, enormously deleterious amounts of prostanoids are released [67,68]. Both constitutive COX-1 and basal COX-2 activities are pivotal for barrier protection [69]. Hence, COX-2 inhibition ought to be selective and partial, with no effect on the constitutive COX-1 and basal COX-2 activity during sepsis therapy. Low doses of CLX were found to blunt the overexpression of COX-2-derived PGE2 in the mucosa of the gut barrier while upholding the homeostatic function of COX-2-derived prostanoids. Such overexpression increases barrier permeability and compromises its tight junctions [67].

The anti-inflammatory activity of CLX has been reported in many previous studies; Du, et al. [70] reported that ability of CLX to inhibit NF-κB activation, and IL-1β and TNF-α release was effective in protecting the cartilage in a rat model of osteoarthritis. In another study, the inhibition of TNF-α, IL-1β, and IL-6 overexpression by CLX in spleen tissues, suppressed splenomegaly, contributed to the development of liver cirrhosis [71]. Furthermore, the suppression of the upregulation of COX-2 expression and, thus, the inflammatory mediators, was evident after the administration of CLX in a rodent model of diabetic nephropathy [72,73].

Many previous studies have reported the antioxidant actions of CLX. The protective effect of CLX in a rat model of tamoxifen-induced liver injury [74], and in a model of cisplatin-induced nephrotoxicity [75], correlated with its ability to reduce MDA levels and increase GSH concentration in examined tissues. Similar effects were also observed in a model of bilateral hindlimb tourniquet IR injury [76]. Such action is critical for preventing multiple organ damage following the induction of sepsis, as MDA levels correlate with the severity of sepsis [77]. The amelioration of oxidative damage that is induced following kidney ischemia/reperfusion was reported in a previous study as a protective action of CLX [78]. Together, these studies illustrated the protective anti-inflammatory and antioxidant effects of IFX and CLX, which are in accordance with our study.

The results of this study, as well as previous studies on the effects of drugs targeting inflammatory mediators during experimental sepsis, introduce a glimpse of hope for sepsis patients. Unfortunately, because human sepsis is very heterogenous, clinical trials examining the effect of different anti-inflammatory therapies have found either no significant benefit or a very small increase in the survival of septic patients. The timing of the treatment also plays an important role in the final outcomes of sepsis; thus, the identification of the exact timepoint(s) at which different treatments should be initiated is a critically important factor that should be the focus of future studies. One limitation of our study is that our treatment can be viewed as an “early intervention” rather than a full therapeutic approach. The two-hours posttreatment protocol presented in the current study reflects a point in which pathological events start to be evident, but it does not reflect the peak of the disease course. As shown in previous reports [79,80], early signs of sepsis, such as bacteremia and elevated levels of serum cytokines, were shown to be evident at timepoints earlier than 6 h after CLP. The difference between clinical sepsis and induced sepsis, as well as the low chance of the early diagnosis of sepsis in humans, lower the translational value of pre-clinical sepsis studies. This raises the need for the identification of efficient markers for sepsis in clinical trials that reflect the stage of sepsis and help design effective treatment approaches.

## 5. Conclusions

The results from this study show that using lower doses of agents that block cytokines or COX-2 had a better potential to improve the outcomes of sepsis. After comparing the effects of both agents used in the current study, we conclude that CLX and IFX, under our experimental conditions, exerted similar protection against sepsis-induced ALI, AKI, and AHI, which was manifested and also reflected as increased survival rates in septic rats in both groups, as compared to the septic untreated rats, with no marked difference among them. Such protective action from both drugs correlated to their anti-inflammatory and antioxidant effects, observed in both of them and with no significant difference in either effect between both agents.

## Figures and Tables

**Table 1:** CLX and IFX ameliorate sepsis-induced changes in pulmonary oxidative and antioxidant profiles in septic rats.

Groups	SOD Activity (u/mg Protein)	GSH Level (nmol/mg Protein)	MDA Level (nmol/mg Protein)	NOX Level (nmol/mg Protein)
**SHAM**	12.96± 0.69	38.38 ± 3.91	0.29 ± 0.01	1.20 ± 0.17
**CLP**	2.87 ± 0.35 #	14.38 ±0.85 #	0.45 ± 0.03 #	3.28 ± 0.33 #
**CLP + CLX**	9.02 ± 1.36 *	28.4 ± 1.91 *	0. 29 ± 0.04 *	1.42 ± 0.28 *
**CLP + IFX**	8.76 ± 0.60 *	28.65 ± 1.40 *	0.32 ± 0.02 *	2.00 ± 0.22 *

ANOVA was used to analyze the results, followed by Tukey–Kramer test for multiple comparisons. For each group, *n* = 6. CLP: cecal ligation and puncture, CLX: celecoxib, IFX: infliximab. # denotes significant difference compared to sham (*p* < 0.05). * denotes significant difference compared to CLP (*p* < 0.05).

**Table 2:** CLX and IFX enhance hepatic oxidative and antioxidant profiles in septic rats.

Groups	SOD Activity (u/mg Protein)	GSH Level (nmol/mg Protein)	MDA Level (nmol/mg Protein)	NOX Level (nmol/mg Protein)
**SHAM**	6.89 ± 0.39	9.74 ± 0.67	0.13 ± 0.01	0.14 ± 0.01
**CLP**	2.00 ± 0.26 #	3.21 ± 0.18 #	0.20 ± 0.01 #	0.20 ± 0.01 #
**CLP + CLX**	3.85 ± 0.14 *	6.77 ± 0.27 *	0.13 ± 0.01 *	0.14 ± 0.01 *
**CLP + IFX**	4.19 ± 0.36 *	7.42 ± 0.83 *	0.15 ± 0.01 *	0.15 ± 0.01 *

One-way ANOVA was used to analyze the results, followed by the Tukey–Kramer test for multiple comparisons. For each group, *n* = 6. CLP: cecal ligation and puncture, CLX: celecoxib, IFX: infliximab. # denotes significant difference compared to sham (*p* < 0.05). * denotes significant difference compared to CLP (*p* < 0.05).

**Table 3:** CLX and IFX enhance renal oxidative and antioxidant profiles in septic rats.

Groups	SOD Activity (u/mg Protein)	GSH Level (nmol/mg Protein)	MDA Level (nmol/mg Protein)	NOX Level (nmol/mg Protein)
**SHAM**	7.41 ± 1.26	11.83 ± 0.79	0.24 ± 0.01	0.4 ± 0.02
**CLP**	0.54 ± 0.11 #	6.15 ± 0.30 #	0.37 ± 0.02 #	1.73 ± 0.31 #
**CLP + CLX**	4.45 ± 1.11 *	10.10 ± 1.42 *	0.26 ± 0.01 *	0.94 ± 0.11 *
**CLP + IFX**	3.72 ± 0.41 *	9.92 ± 0.60 *	0.29 ± 0.01 *	1.00 ± 0.11 *

One-way ANOVA was used to analyze the results, followed by the Tukey–Kramer test for multiple comparisons. For each group, *n* = 6. CLP: cecal ligation and puncture, CLX: celecoxib, IFX: infliximab. # denotes significant difference compared to sham (*p* < 0.05). * denotes significant difference compared to CLP (*p* < 0.05).

## Data Availability

Data is contained within the article.

## Supplementary Materials

The following supporting information can be downloaded at https://www.mdpi.com/article/10.3390/biomedicines10071613/s1, Figure S1:Preliminary studies of survival of CLX (20 mg/kg, p.o), (2 mg/kg, p.o) and (0.5 mg/kg, p.o), Figure S2: Preliminary studies of survival of IFX (7 mg/kg, s.c), Figure S3: Preliminary studies of survival of a group administered a combination of both agents: CLX (0.1 mg/kg, p.o) and IFX (5 mg/kg, s.c).
