# Supplementary material for: Effect of Celecoxib and Infliximab against Multiple Organ Damage Induced by Sepsis in Rats: A Comparative Study
Source: Biomedicines. 2022 Jul 6;10(7):1613. doi: 10.3390/biomedicines10071613 (PMC9312943; doi:10.3390/biomedicines10071613)
Supplement: Supplementary file 1 [file biomedicines-10-01613-s001.zip › biomedicines-1765391-supplementary.pdf]

Supplementary data

Supplementary Figure S1: Preliminary studies of survival of CLX (20 mg/kg, p.o), (2 mg/kg, p.o) and (0.5 mg/kg, p.o)

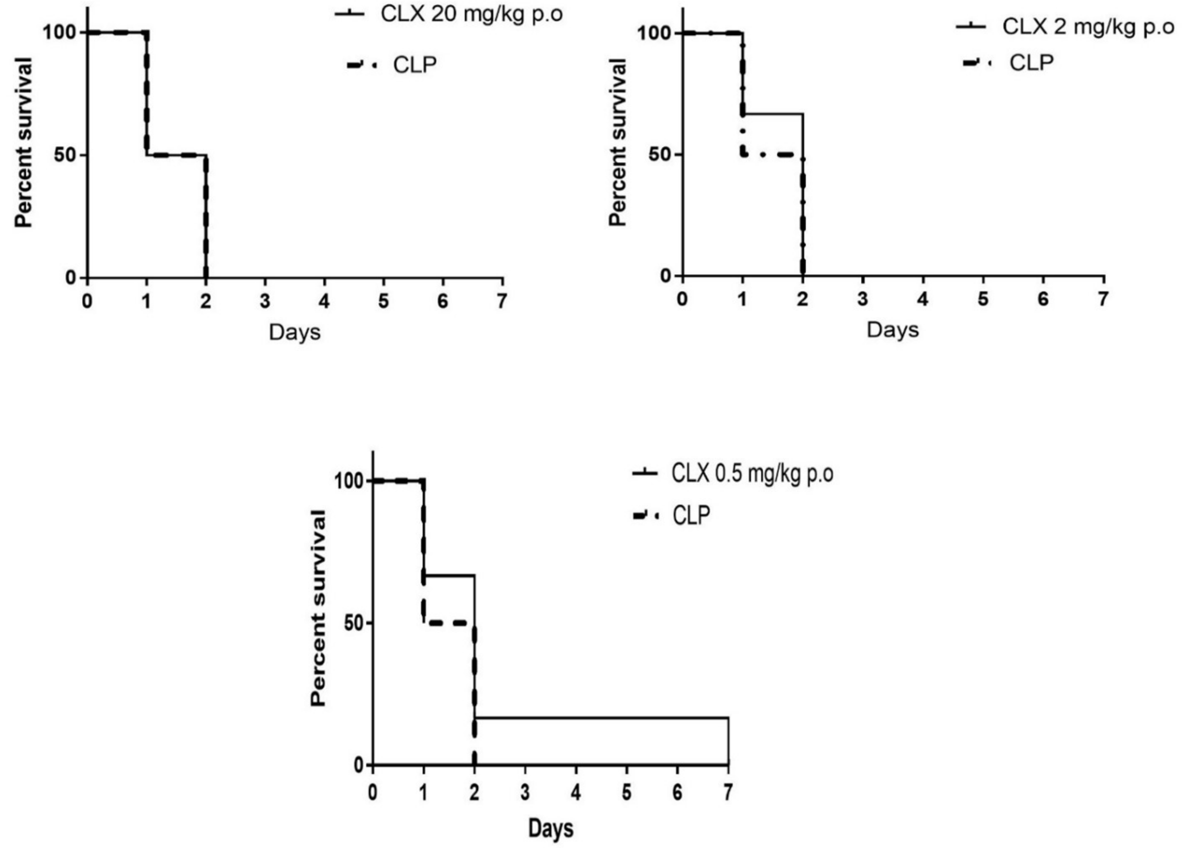

As shown in the supplementary figure1: administration of CLX in a dose of (20 mg/kg, p.o) and (2 mg/kg, p.o) 2 h after performing CLP surgery resulted in (0 %) by the end of the 2nd day. Administration of CLX (0.5 mg/kg, p.o) 2 h following induction of sepsis resulted in (16.6%) survival rate by the end of the 7th day. CLP group showed (0%) survival at the end of the 2nd day.

Supplementary Figure S2: Preliminary studies of survival of IFX (7mg/kg, s.c)

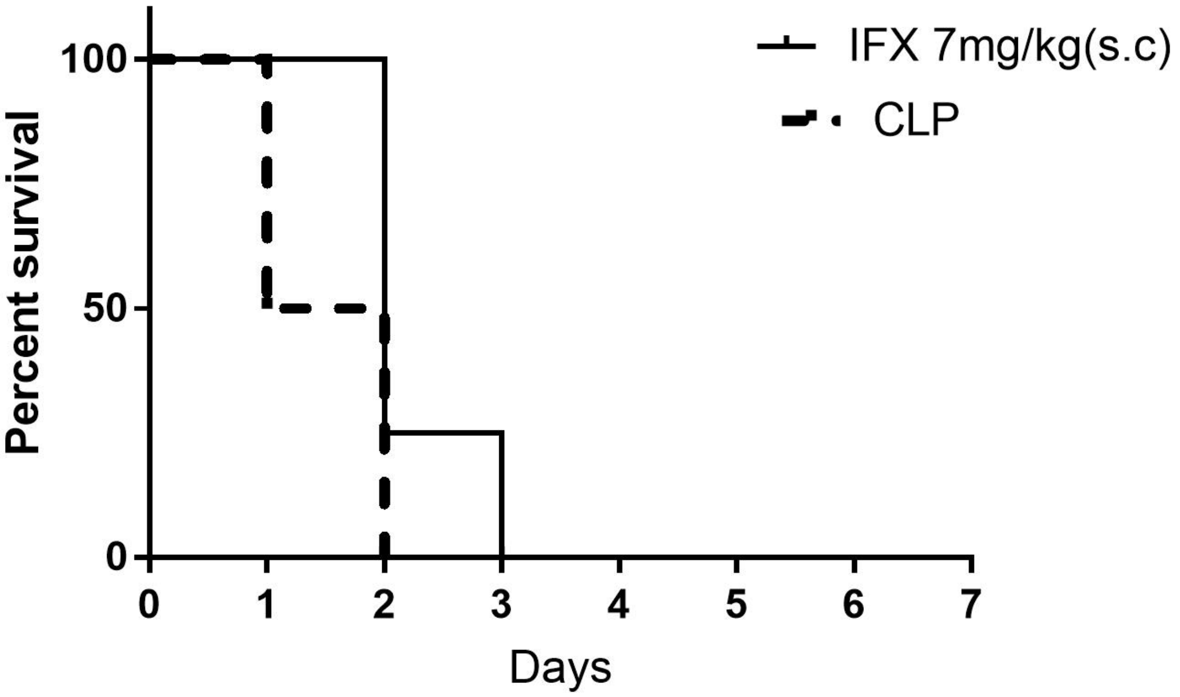

As shown in the supplementary figure2: administration of IFX in a dose of (7 mg/kg, s.c) 2 h after performing CLP surgery showed (25%) survival by the end of the 2nd day and (0%) survival by the end of the 3rd day. CLP group showed (0%) survival at the end of the 2nd day.

Supplementary Figure S3: Preliminary studies of survival of a group administered a combination of both agents: CLX (0.1mg/kg, p.o) and IFX (5mg/kg, s.c)

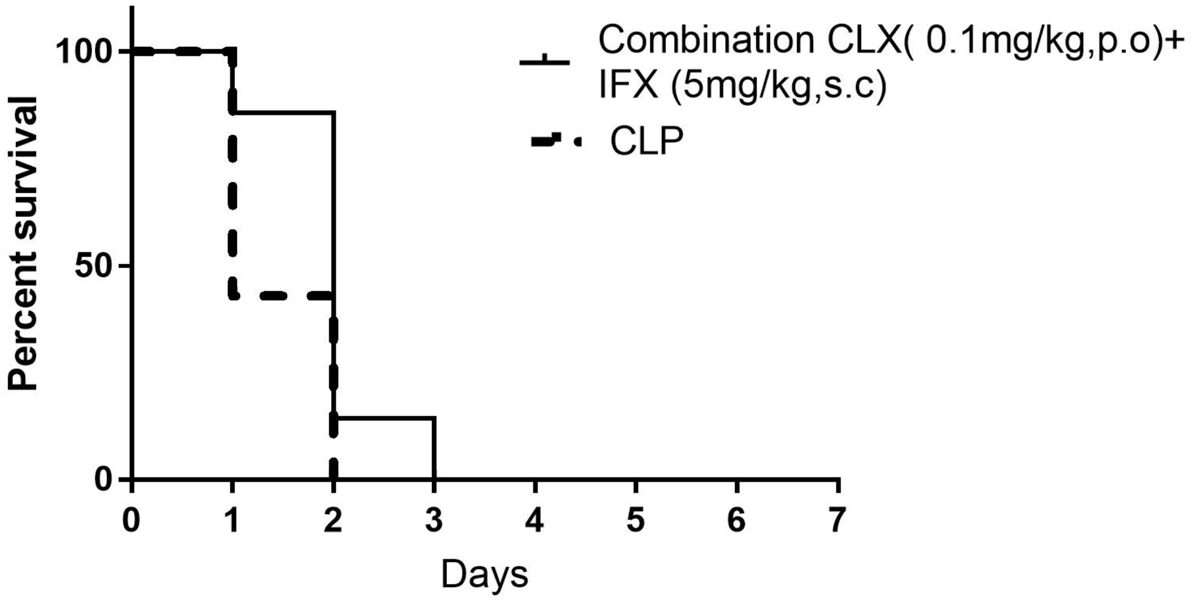

As shown in the supplementary figure3: administration of a combination of CLX (0.1mg/kg, p.o) and IFX (5mg/kg, s.c) 2 h after performing CLP surgery showed (14.3%) survival by the end of the 2nd day and (0%) survival by the end of the 3rd day. CLP group showed (0%) survival at the end of the 2nd day
